# The parapharyngeal vein—an accessory communication between the middle cerebral veins and the internal jugular vein: a case report

**DOI:** 10.1007/s00276-025-03676-y

**Published:** 2025-06-19

**Authors:** Mugurel Constantin Rusu, Răzvan Costin Tudose, Alexandra Diana Vrapciu

**Affiliations:** 1 Division of Anatomy, Department 1, Faculty of Dentistry, “Carol Davila” University of Medicine and Pharmacy, 8 Eroilor Sanitari Blvd, 050474 Bucharest, Romania; 2 Research Department, “Dr. Carol Davila” Central Military Emergency Hospital, 010825 Bucharest, Romania

**Keywords:** Superficial middle cerebral vein, Sphenoidal vein, Internal jugular vein, Parapharyngeal space, Cavernous sinus, Absent maxillary vein, Absent retromandibular vein

## Abstract

**Purpose:**

The superficial middle cerebral vein (SMCV) typically drains into the cavernous sinus, which, in turn, connects to the pterygoid venous plexus via a sphenoidal emissary vein. The latter may course through the foramen ovale. The pterygoid plexus drains in most cases into the retromandibular and facial veins. A peculiar SMCV drainage pathway to the internal jugular vein (IJV) via a parapharyngeal vein was found here.

**Method:**

The anatomic variant reported here was identified by carefully reviewing the archived CT angiogram in a 68-year-old male case.

**Results:**

A double SMCV was found on the right side. The resulting common SMCV trunk passed laterally to the foramen rotundum to empty into the cavernous sinus. A sphenoidal emissary vein joined it, which continued inferiorly through the foramen ovale to the pterygoid plexus. This plexus was connected to a reservoir on the inner side of the lateral pterygoid plate from which a fenestrated parapharyngeal vein left. It had two primary fenestrations, and the proximal one had a fenestrated arm. It reached inferiorly and turned around the external carotid artery. At that level, it received two tributaries: first, the superior thyroid vein and then, the facial vein. The resulting facial-parapharyngeal trunk ended in the IJV. These veins and the carotid arteries, deep to them, were hidden beneath the submandibular gland. The right maxillary vein and the anterior branch of the retromandibular vein were absent.

**Conclusion:**

The parapharyngeal vein may be a direct drainage pathway for the SMCV and the pterygoid plexus. It should therefore be acknowledged and spared during various surgical approaches.

**Supplementary Information:**

The online version contains supplementary material available at 10.1007/s00276-025-03676-y.

## Introduction

The parapharyngeal space (PPS) is an inverted pyramid with the base at the petrous bone and the tip at the hyoid [6, 8]. It is divided into an anterior muscular compartment and a posterior neurovascular one [10].

The superficial middle cerebral vein (SMCV), or the superficial Sylvian vein, courses along the Sylvian fissure and may drain into the cavernous sinus (CS) in 7% of cases (cavernous type of SMCV) [15]. In 12% of cases, it may join the sphenoidal emissary vein (emissary type of SMCV) that further drains into the pterygoid venous plexus (PVP) [15]. The SMCV may be double [4]. The PVP is united with the retromandibular vein by the maxillary veins [2]. Therefore, an extracranial drainage pathway of the SMCV can be established via the PVP. However, the main drainage pathways of the SMCV are intracranial, via the petrosal sinuses to the internal jugular vein (IJV) [4].

A peculiar venous pathway was observed during the retrospective study of veins in a case with previously reported arterial variations [13]. A parapharyngeal vein is used to drain the SMCV into the internal jugular vein (IJV).

## Materials and methods

The archived DICOM file of a 68-year-old male case was studied retrospectively. The scan was routinely performed in a case in which no pathological processes were found to distort the normal vascular anatomy. The research respected the principles of the World Medical Association’s Code of Ethics (Declaration of Helsinki). The responsible authorities (affiliation 2) approved the study (approval no.737/01 November 2024). The CT scans were performed using a 32-slice scanner (Siemens Multislice Perspective Scanner, Forcheim, Germany) with a 0.6 mm collimation and a reconstruction thickness of 0.75 mm, with 50% overlap, for a multiplanar maximum intensity projection. As in other studies, the Horos 3.3.6 (Horos Project, Annapolis, MD, USA) program was used. The findings were checked through two-dimensional reconstructions and three-dimensional volume renderings and measurements were performed.

## Results

**Fig. 1:**
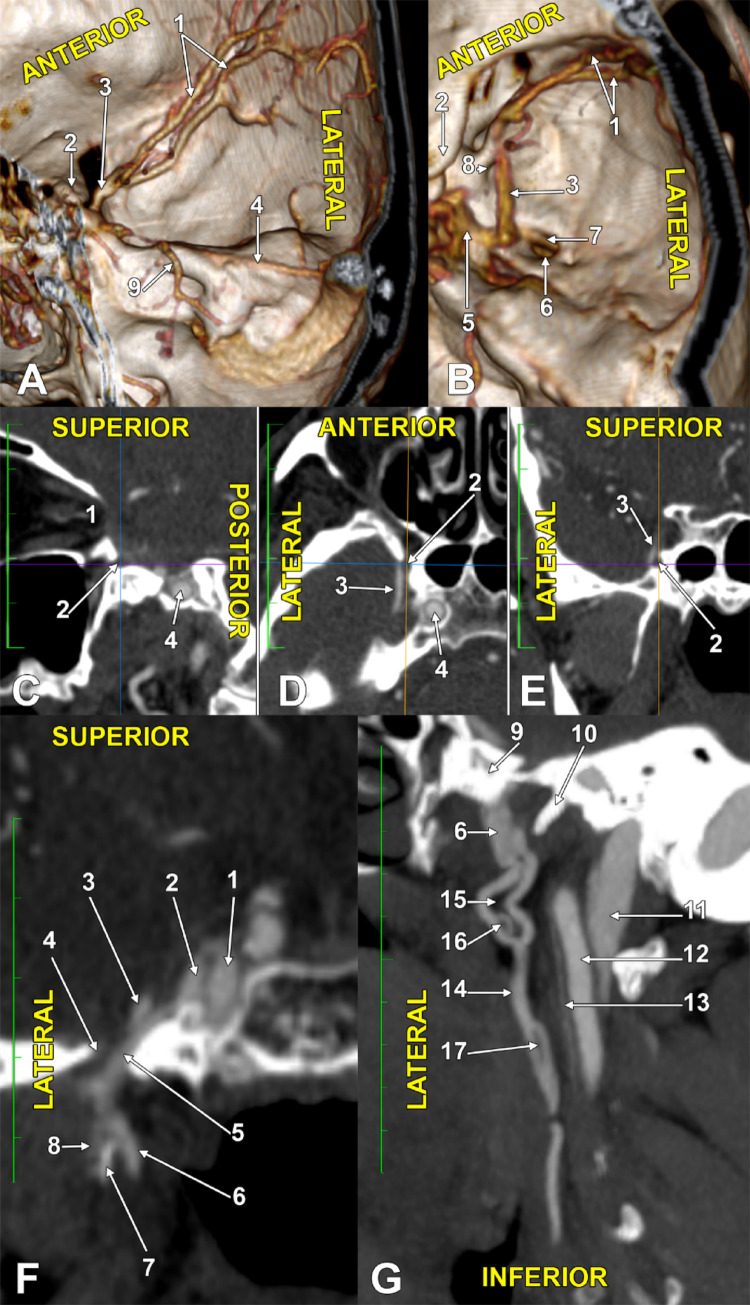
Medial (**A**) and superior (**B**) views of the double SMCV and the resulting common trunk. Three-dimensional volume renderings. (1) double SMCV; (2) anterior clinoid process; (3) common trunk; (4) superior petrosal sinus; (5) cavernous sinus; (6) foramen ovale; (7) sphenoidal emissary vein; (8) foramen rotundum; (9) Dandy’s superior petrosal vein. Correlated sagittal (**C**), axial (**D**) and coronal (**E**) slices through the right foramen rotundum. (1) orbital apex; (2) foramen rotundum; (3) common trunk; (4) internal carotid artery. Connections of the venous reservoir at the upper end of the parapharyngeal vein. Fenestrations of the parapharyngeal vein. **F** Coronal slice. **G** Oblique sagittal slice. (1) internal carotid artery; (2) cavernous sinus; (3) common trunk; (4) foramen ovale; (5) sphenoidal emissary vein; (6) venous reservoir; (7) pterygospinous bar; (8) pterygoid plexus; (9) pterygoid root; (10) sphenoidal spine; (11) internal jugular vein; (12) internal carotid artery; (13) ascending pharyngeal artery; (14) parapharyngeal vein; (15) upper double, anterior and posterior parapharyngeal veins; (16) fenestration of the anterior parapharyngeal vein; (17) inferior slit-like fenestration of the parapharyngeal vein

A double SMCV was found on the right side (Fig. 1A, Online Resource 1). The two veins united to form a common SMCV trunk on the inferior margin of the superior orbital fissure, at 8.04 mm above the foramen rotundum (Fig. 1B, Online Resource 1). That trunk descended onto the greater sphenoidal wing immediately lateral to the foramen rotundum (Fig. 1C, Online Resource 1). It therefore crossed laterally to the maxillary nerve, coursing through that foramen (Fig. 1D, E, Online Resource 1). At 11.31 mm posterior to the foramen rotundum, the common SMCV trunk turned medially and emptied into the CS (Fig. 1B, Online Resource 1). Its preterminal angle was united to a sphenoidal emissary vein that continued inferiorly through the foramen ovale, anteriorly to the mandibular nerve (Fig. 1B, Online Resource 1). The right deep middle cerebral vein was found. It drained posteriorly into the basal vein of Rosenthal and was connected anteriorly with the origin of the common SMCV trunk by a bridging vein (Online Resource 2). The main drainage of the CS was via the superior and inferior petrosal sinuses.

**Fig. 2:**
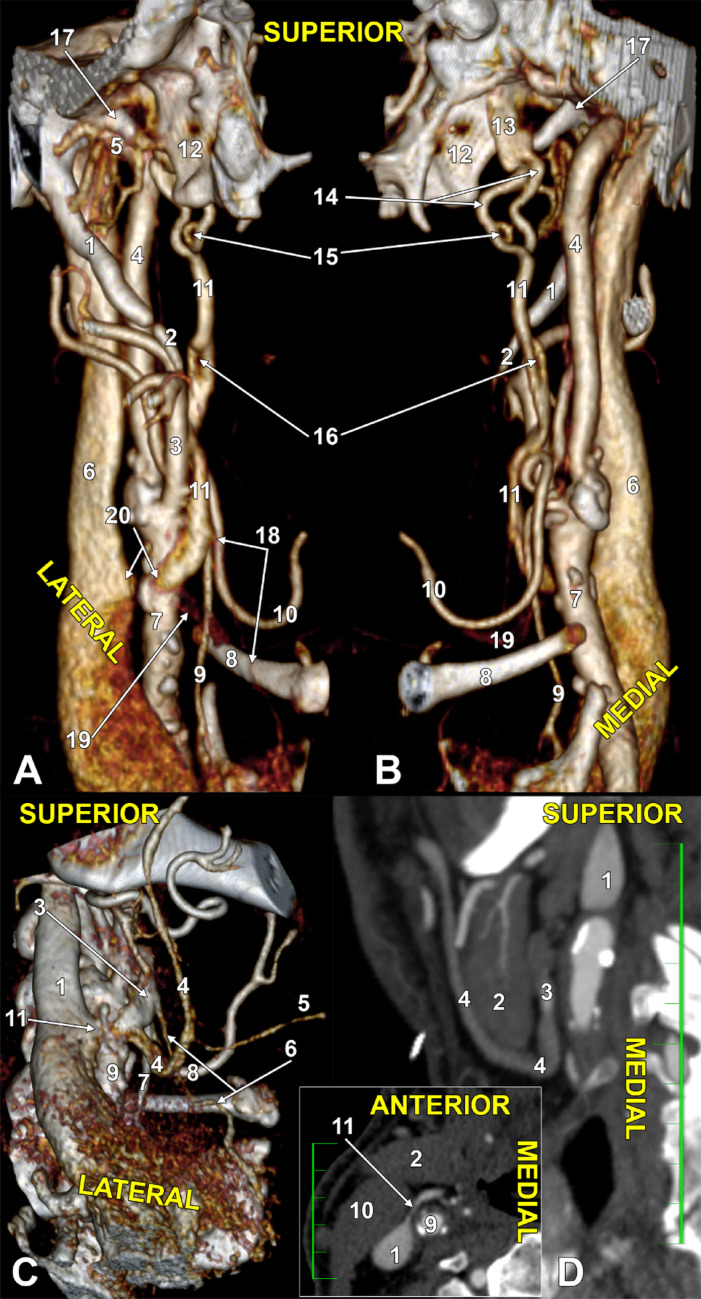
Three-dimensional renderings of the right parapharyngeal vein, antero-lateral view (**A**) and antero-medial view (**B**). (1) styloid process; (2) ossified ceratohyal; (3) external carotid artery; (4) internal carotid artery; (5) pterygoid plexus; (6) internal jugular vein; (7) common carotid artery; (8) greater hyoid horn; (9) superior thyroid artery; (10) lingual artery; (11) parapharyngeal vein; (12) lateral pterygoid plate; (13) venous reservoir; (14) upper double, anterior and posterior parapharyngeal veins; (15) fenestration of the anterior parapharyngeal vein; (16) slit-like fenestration of the parapharyngeal vein; (17) sphenoidal spine; (18) superior thyroid vein; (19) facial vein; (20) facial-parapharyngeal trunk. Venous anatomy of the terminal segment of the right parapharyngeal vein. **C** Three-dimensional rendering, infero-lateral view. **D** Coronal slice through the submandibular gland, anterior view. Inset: axial slice through the facial-parapharyngeal trunk, inferior view. (1) internal jugular vein; (2) submandibular gland; (3) parapharyngeal vein; (4) facial vein; (5) submental vein; (6) superior thyroid vein; (7) superior thyroid artery; (8) lingual artery; (9) common carotid artery; (10) sternocleidomastoid muscle; (11) facial-parapharyngeal trunk

The sphenoidal emissary vein connected inferiorly to the PVP on the deep side of the lateral pterygoid muscle and, respectively, to a 5.28 mm large and 14.5 mm long reservoir on the medial side of the lateral pterygoid plate (Fig. 1, Online Resource 1). That venous reservoir and the PVP were interconnected above a pterygospinous bar (Fig. 1F). From that venous reservoir, a consistent parapharyngeal vein continued inferiorly in the PPS, between the medial pterygoid muscle and the pharyngeal wall, antero-medially to the internal carotid artery (Figs. 1G and 2A, B).

In the proximal 14.9 mm of its course, the parapharyngeal vein was double; on the anterior branch, a two-millimetre fenestration was found (Online Resource 1). At 27 mm distally to the first fenestration, the parapharyngeal vein presented a slit-like second fenestration of 6.3 mm long (Figs. 1G and 2A, B, Online Resource 1). After crossing posteriorly the stylopharyngeus muscle, it crossed the medial wall of the external carotid artery, immediately above the superior loop of the lingual artery. It further descended at 3.4 mm anterior to the external carotid artery.

The distance between the hyoid and the mandible was 4.86 cm. The right submandibular gland filled that space almost completely and was applied to the anterior margin of the sternocleidomastoid muscle. Deep to both of these, the carotid bifurcation was at 26.4 mm superior to the greater hyoid horn. The parapharyngeal vein crossed over the common carotid artery at 7.9 mm inferior to its bifurcation. It was therefore deep to the submandibular gland. Over the gland descended the facial vein that looped inferiorly to the gland, received the submental vein, and emptied into the parapharyngeal vein. The superior thyroid vein ascended at 13.4 mm anterior to the superior thyroid artery, crossing the greater hyoid horn laterally. The superior thyroid vein continued deep to the submandibular gland and ended into the parapharyngeal vein at 19.7 mm above the greater hyoid horn (Figs. 1 and 2). It resulted in a terminal facial-parapharyngeal venous trunk, 8.7 mm, that ended into the IJV at 10.9 mm inferior to the carotid bifurcation, deep to the anterior margin of the sternocleidomastoid muscle (Figs. 1 and 2, Online Resource 1).

The right maxillary vein was absent. Also, the anterior branch of the retromandibular vein was absent. The retromandibular vein resulted from the superficial temporal vein and continued as the external jugular vein.

On the opposite side, a single SMCV was found draining into the CS. Both the left deep middle cerebral vein and the left basal vein were connected to the SMCV.

## Discussion

During the embryogenesis of the head and neck veins, the ventral pharyngeal vein first drains into the common cardinal vein but later drains into the precardinal vein, which will further form the IJV [1]. Typically, the primitive maxillary vein joins the ventral pharyngeal vein and thus the facial vein results [1]. It will further connect the retromandibular vein [1]. In our case, the PVP was not connected to the retromandibular vein as expected. Nor was it connected to the facial vein. It is therefore reasonable to speculate that during embryogenesis, the primitive maxillary vein did not connect to the retromandibular vein but kept its connection with the precardinal vein. That connection elongated and became the parapharyngeal vein. This vein is a novel accessory venous communication between the SMCV and the IJV.

The parapharyngeal vein in our case modified the drainage pathway of the PVP and, therefore, of the SMCV because the PVP was not connected to the retromandibular vein. Instead, the PVP drained into the IJV via the parapharyngeal vein.

According to its drainage, the SMCV may be of cavernous, sphenobasal, or sphenopetrosal types, or absent [14]. The sphenobasal type indicates a SMCV coursing through the foramen ovale [14], as in the present study. This type of SMCV was found to have a prevalence of 18.8% [14]. In a typical case, the sphenobasal SMCV uses the path of a sphenoidal emissary vein to empty into the PVP on the deep side of the lateral pterygoid muscle. Here, we found a sphenobasal SMCV draining via the parapharyngeal vein into the IJV. Suzuki and Matsumoto (2000) described the cavernous and emissary types of SMCV [15]. In just 3% of cases, they found multiple drainage pathways of the SMCV but they did not detail the specific combinations [15]. We found here a double SMCV draining equally into the cavernous sinus and the sphenoidal emissary vein, indicating a combined type that should be added to these authors’ types, specifically a cavernous-emissary combined type. When double and combined paths of drainage in the SMCV territory occur, they ensure safe drainage of that territory.

We found that on both sides, the SMCVs were connected to the deep middle cerebral veins. Moreover, on the left, the basal vein was also connected to the SMCV. Rhoton (2002) described that „the middle cerebral vein, the anterior segment of the basal vein, or their tributaries may be connected by a bridging vein to the sphenoparietal or cavernous sinus” [12]. We propose here a novel possibility regarding these connections: the deep middle cerebral vein and/or the basal vein may connect anteriorly to a cavernous type of the superior middle cerebral vein (SMCV).

The emissary vein of the foramen ovale is a major emissary vein in the middle cranial fossa that runs inferiorly through that foramen and joins the PVP and/or the pharyngeal plexus [3]. We did not find a morphologically defined venous pharyngeal plexus here. Still, we found instead a longitudinal fenestrated vein that we termed „parapharyngeal vein” as it did not originate within the pharyngeal wall and coursed through the PPS.

In our case, the topography of the submandibular gland was modified. It reached over the carotid triangle’s contents and hid them from an eventual direct dissection. Surgeons or interventionists should document or anticipate this topographic possibility when approaching the carotid arteries. Nevertheless, the parapharyngeal vein we found and report here is a previously unrecognised vein that distorts the expected venous morphology in which the common facial vein crosses over the submandibular gland, receives the superior thyroid vein, and empties into the IJV at an acute angle, opening superiorly. In our case, that venous angle was formed by a facial-parapharyngeal trunk, and the upper end of the superior thyroid vein was inserted into the parapharyngeal vein proximally to the end of the facial vein. The loop of the facial vein immediately beneath the submandibular gland could also be a disorienting landmark for surgeons. Moreover, the terminal segment of the parapharyngeal vein should cross the hypoglossal nerve when crossing the external carotid and lingual arteries and before ending into the IJV. This puts the neurovascular content of the carotid triangle at risk, especially when the submandibular gland covers it and the surgical field is modified.

A high carotid bifurcation was found in this case. A recent study demonstrated that carotid bifurcations were located above the greater hyoid horn in 19.73% of 294 carotid bifurcations [7], indicating that this variation is not rare. In such cases, a high carotid bifurcation results in a modified topography of the carotid system’s branches.

This novel venous route reported here is clinically and surgically significant. The parapharyngeal vein bypasses the usual collateral-rich pathways, such as the CS and PVP, and channels venous outflow through a single, pressure-sensitive route. In the supine position, where the IJV is the primary drainage pathway, any compression, such as that caused by head positioning or central line placement, may compromise cerebral outflow and elevate intracranial pressure [5]. Surgically unrecognised variants like this pose a risk during neck dissection, skull base approaches, or transvenous procedures, where inadvertent injury may cause significant venous bleeding or postoperative infarction due to the lack of alternative drainage routes [9]. An unrecognised parapharyngeal vein is exposed to surgical risks and should be known and discriminated before surgical dissections or interventional procedures. Preoperative venous mapping is therefore substantial when planning interventions near the PPS or jugular system. Importantly, better identification of such optional venous pathways is critical for accurately assessing pre- and post-embolisation hemodynamics in brain arteriovenous malformations, where unexpected drainage patterns can influence treatment planning and outcomes [11].

A significant limitation of this report of a novel anatomic variation is that it could not establish the prevalence of the parapharyngeal vein. This will be included as a specific aim in further research. The absence of direct contrast injection into the venous system limits the ability to precisely determine venous connections and flow direction.

In conclusion, the parapharyngeal vein may be regarded as a direct drainage pathway for the SMCV and the PVP. Therefore, it should be acknowledged, identified, and spared during specific surgical approaches. This is because it increases the risk of bleeding during approaches to the parapharyngeal space and modifies the surgical landmarks of the neurovascular content of the carotid triangle. When the parapharyngeal vein is associated with an anomalous arterial topography and the submandibular gland is lowered, the surgical approaches should be performed cautiously.

## Electronic supplementary material

Below is the link to the electronic supplementary material.

Supplementary Material 1

Supplementary Material 2

## Data Availability

The datasets used and analysed during the current study are available from the corresponding author upon reasonable request.
